# The effect of electrical conductivity of myocardium on cardiac pumping efficacy: a computational study

**DOI:** 10.1186/s12938-016-0295-6

**Published:** 2017-01-10

**Authors:** Ana Rahma Yuniarti, Ki Moo Lim

**Affiliations:** Department of IT Convergence Engineering, Kumoh National Institute of Technology, 61 Daehak-ro, Gumi, 39177 South Korea

**Keywords:** Conduction velocity, Electromechanical model, Cardiac pumping efficacy

## Abstract

**Background and aims:**

The existence of non-excitable cells in the myocardium leads to the increasing conduction non-uniformity and decreasing myocardial electrical conductivity. Slowed myocardial conduction velocity (MCV) believed to enhance the probability of cardiac arryhthmia and alter the cardiac mechanical pumping efficacy, even in sinus rhythm. Though several studies on the correlation between MCV and cardiac electrical instabilities exist, there has been no study concerning correlation or causality between MCV and cardiac mechanical pumping efficacy, due to the limitation in clinical methods to document and evaluate cardiac mechanical responses directly. The goal of this study was to examine quantitatively the cardiac pumping efficacy under various MCV conditions using three-dimensional (3D) electromechanical model of canine’s failing ventricle.

**Methods:**

The electromechanical model used in this study composed of the electrical model coupled with the mechanical contraction model along with a lumped model of the circulatory system. The electrical model consisted of 241,725 nodes and 1,298,751 elements of tetrahedral mesh, whereas the mechanical model consisted of 356 nodes and 172 elements of hexahedral mesh with Hermite basis. First, we performed the electrical simulation for five different MCV conditions, from 30 to 70 cm/s with 10 cm/s interval during sinus pacing. Then, we compared the cardiac electrical and mechanical responses of each MCV condition, such as the electrical activation time (EAT), pressure, volume, and energy consumption of the myocardium. The energy consumption of the myocardium was calculated by integrating ATP consumption rate of each node in myofilament model.

**Results:**

The result showed that under higher MCV conditions, the EAT, energy consumption, end diastolic and systolic volume are gradually decreased. Meanwhile, the systolic pressure, stroke volume, stroke work, and stroke work to ATP are increased as the MCV values increased. The cardiac functions and performances are more efficient under higher MCV conditions by consuming smaller energy (ATP) while carrying more works.

**Conclusion:**

In conclusion, this study reveals that MCV has strong correlation with the cardiac pumping efficacy. The obtained results provide useful information to estimate the effect of MCV on the electro-physiology and hemodynamic responses of the ventricle and can be used for further study about arrhythmogeneis and heart failure.

## Background

Action potential (AP) propagates from one myocyte to the next myocyte through gap junctions. However, there are not only myocytes exist in the myocardium but also non-excitable cells such as collagenous strands, blood vessels, and fibroblasts. Those non-excitable cells affect electrical properties by increasing conduction non-uniformity and decreasing myocardial electrical conductivity. Myocardial conduction velocity (MCV) varies from 30 to 70 cm/s depending on the level of non-uniformity. Slowed MCV is associated with an increased risk of re-entrant excitation, predisposing to cardiac arrhythmia [1] and will also change cardiac mechanical pumping efficacy, even in sinus rhythm.

Quantitative analysis of the effect of MCV on cardiac electrophysiology and mechanical pumping function is highly important for further research of cardiac arrhythmia and heart failure. Though several studies on the correlation between MCV and cardiac electrical instabilities exist [2–5], there has been no study concerning correlation or causality between MCV and cardiac mechanical pumping efficacy. This is because experimental methods to document and evaluate cardiac mechanical responses directly, such as cardiac output, myocardial tension and strain generation throughout the ventricular volume, and cardiac electromechanical interaction, are hampered by low spatiotemporal resolution. Computational modeling is an alternative approach that overcomes this limitation.

We have previously developed a three-dimensional (3D) electromechanical model of failing canine ventricles along with a lumped model of the circulatory system [6–8]. The goal of this study is to use the computational model of the heart to examine the cardiac mechanical responses under various MCV conditions, and determine any causality between MCV and cardiac pumping efficacy.

## Methods

### Computational model of the heart

**Fig. 1:**
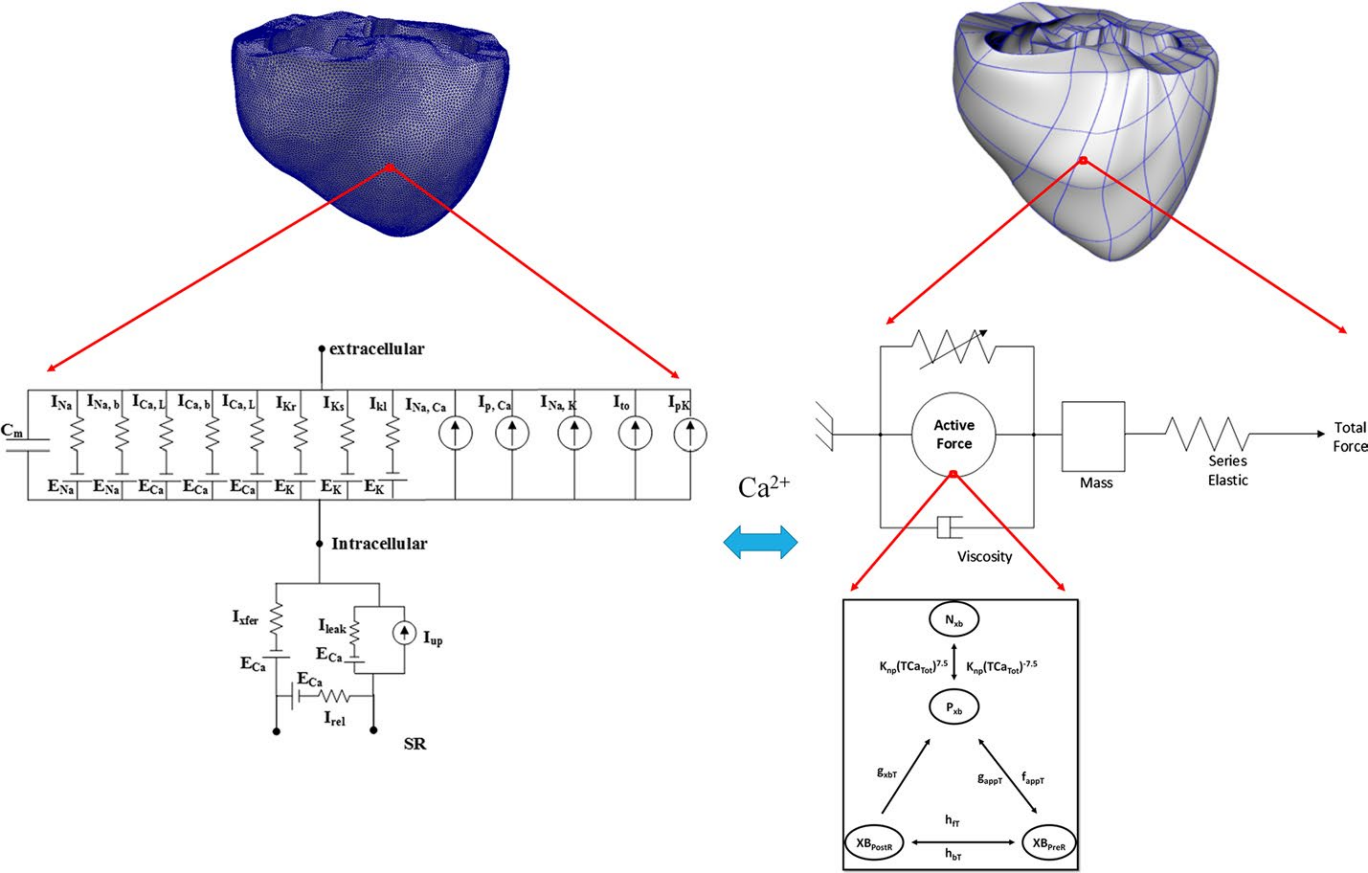
Schematic representation of ventricular electromechanical modeling. *Left side* is electrical components and *right side* is mechanical components. The electrical components consist of cell membrane Cm as a capacitive component connected in parallel with variable resistances and batteries, representing the ionic currents and pumps. $I_{\rm{Na}}$ is rapid inward Na^+^ current, $I_{\rm{Na,b}}$ $I_{\rm{Ca,b}}$ is background Na^+^ and Ca^2+^ current, $I_{\rm{Ca,L}}$ is L-type Ca^2+^ current, $I_{\rm{Kr}}$ is rapid delayed rectifier ${\rm{K^+}}$ current, $I_{\rm{Ks}}$ is slow delayed rectifier ${\rm{K^+}}$ current, $I_{\rm{K1}}$ is inward rectifier ${\rm{K^+}}$ current, $I_{\rm{Na,Ca}}$ is Na^+^–Ca^2+^ exchanger current, $I_{\rm{p,Ca}}$ is Ca^2+^ pump current, $I_{\rm{Na,K}}$ is Na^+^–${\rm{K^+}}$ pump current, $I_{\rm{to}}$ is transient outward ${\rm{K^+}}$ current, $I_{\rm{p,K}}$ is ${\rm{K^+}}$ pump current, $I_{leak}$ is Ca^2+^ released current from SR, $I_{up}$ is SR Ca^2+^ uptake current, $I_{rel}$ is Ca^2+^ induced-Ca^2+^ released current, $E_{\rm{Na}}$ is Na^+^ pump, $E_{\rm{Ca}}$ is Ca^2+^ pump, and $E_K$ is ${\rm{K^+}}$ pump. The mechanical component represented by the myofilament model. $N_{xB}$ and $P_{xB}$ are non-permissive and permissive confirmation of regulatory proteins, $XB_{PreR}$ and $XB_{PostR}$ are pre-rotated and post-rotated states of myosin head-binding

To construct the computational model for this study, we employed the existing 3D electromechanical model, combined with a lumped model of cardiovascular system [6–8]. The electromechanical model consists of finite element electrical and mechanical model, which describe the behavior and interaction between electrical activation and mechanical contraction of the ventricle. Both of electrical and mechanical model was reconstructed from high resolution magnetic resonance (MR) and diffusion tensor (DT) MR imaging of the failing canine ventricle. The methodology to reconstruct the model from image has been described elsewhere [8]. The whole schematic for this model can be seen in Fig. 1.

### Description of electrical model

The 3D electrical model was constructed through finite element of tetrahedral mesh, consists of 241,725 nodes and 1,298,751 elements. The mesh has the characteristic of realistic heart compartments including the endocardium, mid-myocardium, and epicardium, as well as purkinje fibers. The model mimics the propagation of AP in cardiac tissue using an electrical conduction equation, derived from continuum mechanics. The equation describes the continuum characteristic of the current flow through cardiomyocytes which are connected electrically via conductive gap junction. The current flow in the ventricular tissue was driven by active ion exchange across the cell membrane. Many researchers have made a great contribution in developing mathematical models of these ionic properties in the cardiac myocyte [9–14]. In this study, we adopted the ionic myocyte model from Ten Tusscher et al. [12]. The model describes the cell membrane as a capacitive component connected in parallel with the resistors and batteries, representing the ionic current flows from cell to cell due to low-resistance gap junction, pumps, and transporters. The electrical behavior in a single cell can be described as follows:

1 $$\begin{aligned} \frac{dV}{dt}&= - \frac{I_{ion} + I_{stim}}{C_m} \end{aligned}$$

where *V* (mV) is the membrane potential of one cell, *t* (ms) is time, $I_{ion}$ (*pA*/*pF*) is the total transmembrane ionic current, $I_{stim}$ (*pA*/*pF*) is the total external stimulus current, and $C_m$ (μF/cm^2^) is cell capacitance per unit surface area. A negative value represents a transmembrane at rest.

While the equation for electrical behavior in 3D cardiac tissue is represented by:

2 $$\begin{aligned} \frac{dV}{dt}&= -\frac{I_{ion} + I_{stim}}{C_m}+\frac{1}{\rho _x S_x C_m}\frac{\partial ^2V}{\partial x^2}+\frac{1}{\rho _y S_y C_m} \frac{\partial ^2V}{\partial y^2}+\frac{1}{\rho _z S_z C_m} \frac{\partial ^2V}{\partial z^2} \nonumber \\ \end{aligned}$$

where $\rho _x$, $\rho _y$, and $\rho _z$ ($\Omega$ cm) are the cellular resistivity in the *x*, *y*, and *z* directions, and $S_x$, $S_y$, and $S_z$ are the surface-to-volume ratio in the in the *x*, *y*, and *z* directions. The total transmembrane ionic current, $I_{ion}$, is given by following equation:

3 $$\begin{aligned} I_{ion}&= I_{\rm{Na}}+I_{\rm{K1}}+I_{\rm{to}}+I_{\rm{Kr}}+I_{\rm{Ks}}+I_{\rm{CaL}}+I_{\rm{NaCa}}+I_{\rm{NaK}}+I_{\rm{pCa}}+I_{\rm{pK}}+I_{\rm{bCa}}+I_{\rm{bNa}} \nonumber \\ \end{aligned}$$

where $I_{\rm{Na}}$ is rapid inward Na^+^ current, $I_{\rm{K1}}$ is inward rectifier ${\rm{K^+}}$ current, $I_{\rm{to}}$ is transient outward ${\rm{K^+}}$ current, $I_{\rm{Kr}}$ is rapid delayed rectifier ${\rm{K^+}}$ current, $I_{\rm{Ks}}$ is slow delayed rectifier ${\rm{K^+}}$ current, $I_{\rm{Ca,L}}$ is L-type Ca^2+^ current, $I_{\rm{NaCa}}$ is Na^+^–Ca^2+^ exchanger current, $I_{\rm{NaK}}$ is Na^+^-${\rm{K^+}}$ pump current, $I_{\rm{pCa}}$ is Ca^2+^ pump current, $I_{\rm{pK}}$ is ${\rm{K^+}}$ pump current, $I_{\rm{bCa}}$ is background Ca^2+^ current, and $I_{\rm{bNa}}$ is background Na^+^ current. The more details of model parameters can be found in ten Tusscher et al. [12].

### Description of EC coupling model

The cardiac muscle contracts via excitation–contraction (EC) coupling. EC coupling can be described as the process of converting an electrical excitation into a force generation, resulting in the occurrence of contraction of the heart. EC coupling occurs, as the AP depolarized through the heart, activating the release of calcium (Ca^2+^) from the sarcoplasmic reticulum (SR), which causes the Ca^2+^ concentration in the cytoplasm to increase. The Ca^2+^ then binds to troponin C, inducing a deformation of troponin and subsequently moves the tropomyosin away from the actin-binding sites. This removal of tropomyosin allows the myosin head to pull the actin filament toward the center of sarcomere, forming the cross-bridge cycles and triggering contraction.

To remodel EC coupling in our study, we incorporated the electrical model as described in the previous section, with the mechanical model of myocyte filament from Rice et al. [13] using the calcium dynamic, which is also referred to Ten Tusscher model [12]. The calcium dynamic serves as the input to the myocyte filament model, as illustrated by the Ca^2+^ binding to troponin C and cross-bridge cycling, given by:

4 $$\begin{aligned} \frac{dCa_{tot}}{dt}&= - \frac{V_c}{V_{sr}}(-I_{leak}+I_{up}-I_{rel}) \end{aligned}$$

where $Ca_{tot}$ (mM) is the total calcium in the SR, $V_c$ (μm^3^) is the cytoplasmic volume, $V_{sr}$ (μm^3^) is the SR volume, $I_{leak}$ (*pA*/*pF*) is the leakage current from SR to cytoplasm, $I_{up}$ (*pA*/*pF*) is the pumping current required to return the calcium to the SR, and $I_{rel}$ (*pA*/*pF*) is the calcium-induced calcium released current. For details, see Ten Tusscher et al. [12].

The mechanical model illustrates the active contraction and deformation of ventricles in the form of myofilament dynamics, including descriptions of attachment and detachment of cross-bridges and their elastic properties. The active contraction of the ventricles is a result of the active tension generated by the myofilament dynamics model of Rice et al. [13]. The active tension itself depended on the ratio of fiber length before and after deformation, and its temporal derivative of the ventricular cell. The deformation of the ventricles was derived from the equations of passive cardiac mechanics, with the myocardium assumed to be an orthotropic, hyperelastic, and nearly incompressible material, with the passive mechanical properties defined by an exponential strain-energy function [15]. The simulation of ventricular contraction was performed by simultaneously solving the myofilament model equations with the passive cardiac mechanics equations on the mechanical mesh. This mechanical mesh consists of 356 nodes and 172 hexahedral finite elements with a Hermite polynomial basis.

### Description of a lumped model of circulatory system

To simulate the ventricular hemodynamics, we coupled the finite element electromechanical model with a lumped model of the systemic and pulmonic circulations based on Kerckhoffs et al. [16]. Both of the circulation systems were modeled as two lumped Windkessel compartments in series, one compartment for arterial and capillary blood, and one for venous blood. Each compartment was indicated by a resistance and compliance parameter. Resistance (i.e resistor) expresses flow resistance inside blood vessels due to viscosity, and compliance (i.e capacitor) determines the pressure–volume (PV) relationship for each segment through the fluid analog of the law of capacitance. All segments were modeled with linear PV relationships. Using conservation of mass law (for incompressible blood leading to conservation of volume), the volume change of a Windkessel segment was determined by inflow minus outflow. The circuit diagram of this circulatory system can be seen in Fig. 1, along with the finite element of mechanical model. Next, to implement the failing ventricle, we decreased 10% compliances of vascular system [6] in order to mimic the atherosclerosis and hypertensive condition.

### Simulation Protocol

To achieve the goals of this study, first, we performed the electrical simulation for 3 s using sinus pacing with one cycle length of 600 ms. We simulated several MCV cases: 30, 40, 50, 60, and 70 cm/s for this study. This variation are chosen first, by setting the normal MCV as 70 cm/s, according to the previous study from ten Tusscher et al. [12], where the maximum planar coduction was achieved. Additionally, this normal MCV value is chosen because it has the same value with the study reported by Jongsman and Wilders [17] for longitudinal conduction velocity in a linear cable of PB cell model as well as the experimental data of Taggart et al. [18]. Then, to simulate various MCV level, we reduced MCV value from normal condition (i.e 70 cm/s) with 10 cm/s interval to 30 cm/s. To obtain the 70 cm/s MCV condition in this study, we set the cellular resistivity, $\rho$, in the electrical model into 162 $\omega {\rm{cm}}$, follwing ten Tusscher et al. [12]. Then, we increased $\rho$ 1.25 times to obtain 60 cm/s MCV condition, 1.67 times to obtain 50 cm/s MCV condition, 2.5 times to obtain 40 cm/s MCV condition, and 3.3 times to obtain 30 cm/s MCV condition. The *MCV* (cm/s) values used for this study were determined by measuring the distance *d*(cm) between two points in the ventricle and dividing by the time *t*(*s*) taken to complete the activation of ventricle, as given by:

5 $$\begin{aligned} MCV&= \frac{d}{t} \end{aligned}$$

We took last cycle data (i.e., from 2.4 to 3 s) of the electrical simulation results to generate the electrical activation time (EAT). EAT is time of cellular depolarization, which determined the instants at which the combined ionic and cardiac myofilament model in the mechanics component was stimulated. The EAT distribution is following the experimental data of Durrer et al. [19] by implementing sinus rhythm pacing, where stimulation initiated from the endocardium at specific timings and locations to replicate electrical activation originating from the Purkinje fibers. The EAT was then coupled with intracellular Ca^2+^ cycling, which served as the inputs for mechanical contraction model, as the instances when the myofilament model is activated. Next, we ran the mechanical contraction simulation for 32 cycles to obtain steady responses. Lastly, we took the end of the cycle data of the cardiac mechanical responses to analyze the cardiac pumping efficacy. These responses included pressure, volume, energy consumption in the form of ATP, and contractility of ventricle. The energy consumption of the myocardium in this study was calculated by integrating the ATP consumption of each node of the ventricles with time for one cycle (600 ms). The ATP consumption is derived from the contractile ATP consumption rate in the myofilament model of Rice et al. [13], as follows:

6 $$\begin{aligned} ATP\,consumption\,rate = g_{xbT} \times SOVF_{Thick} \end{aligned}$$

where $g_{xbT}$ is the cross-bridge detachment rate, and $SOVF_{Thick}$ is the single overlap function of the thick filaments.

## Results

### Electrophysiological responses

**Fig. 2:**
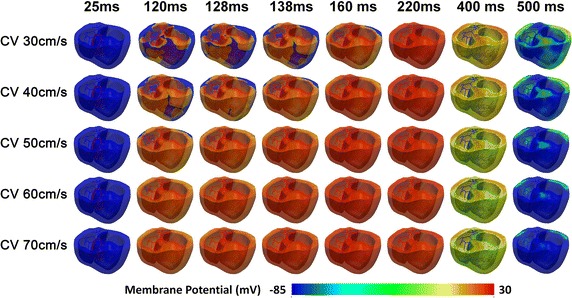
Transmural distribution of membrane potential in time series under various conduction velocities. Conduction velocity variations are 30, 40, 50, 60, and 70 cm/s. CV indicates conduction velocity

Figure 2 represents the transmural distribution of the membrane potential during one cycle of sinus pacing. Because the conduction velocity through Purkinje fibers is 200 cm/s in all conditions [19], ventricular stimulation was initiated at 25ms in all cases. The entire ventricle was depolarized at 120 ms in 70 cm/s MCV case, 128 ms in 60 cm/s MCV case, 138 ms in 50 cm/s MCV case, 160 ms in 40 cm/s MCV case, and 220 ms in 30 cm/s MCV case. The ventricle was repolarized in same order as depolarization. The membrane potential scale was set from −85 to 30 mV. Depolarization waves were observed starting from the endocardial tissue. This is because the terminal node of Purkinje fibers is embedded in the endocardium. In all cases, depolarization wave lasted longest in mid-myocardium region. This is because AP duration (APD) is longest in mid-myocardium among the three types of myocardial tissue: endocardium, mid-myocardium, and epicardium.

**Fig. 3:**
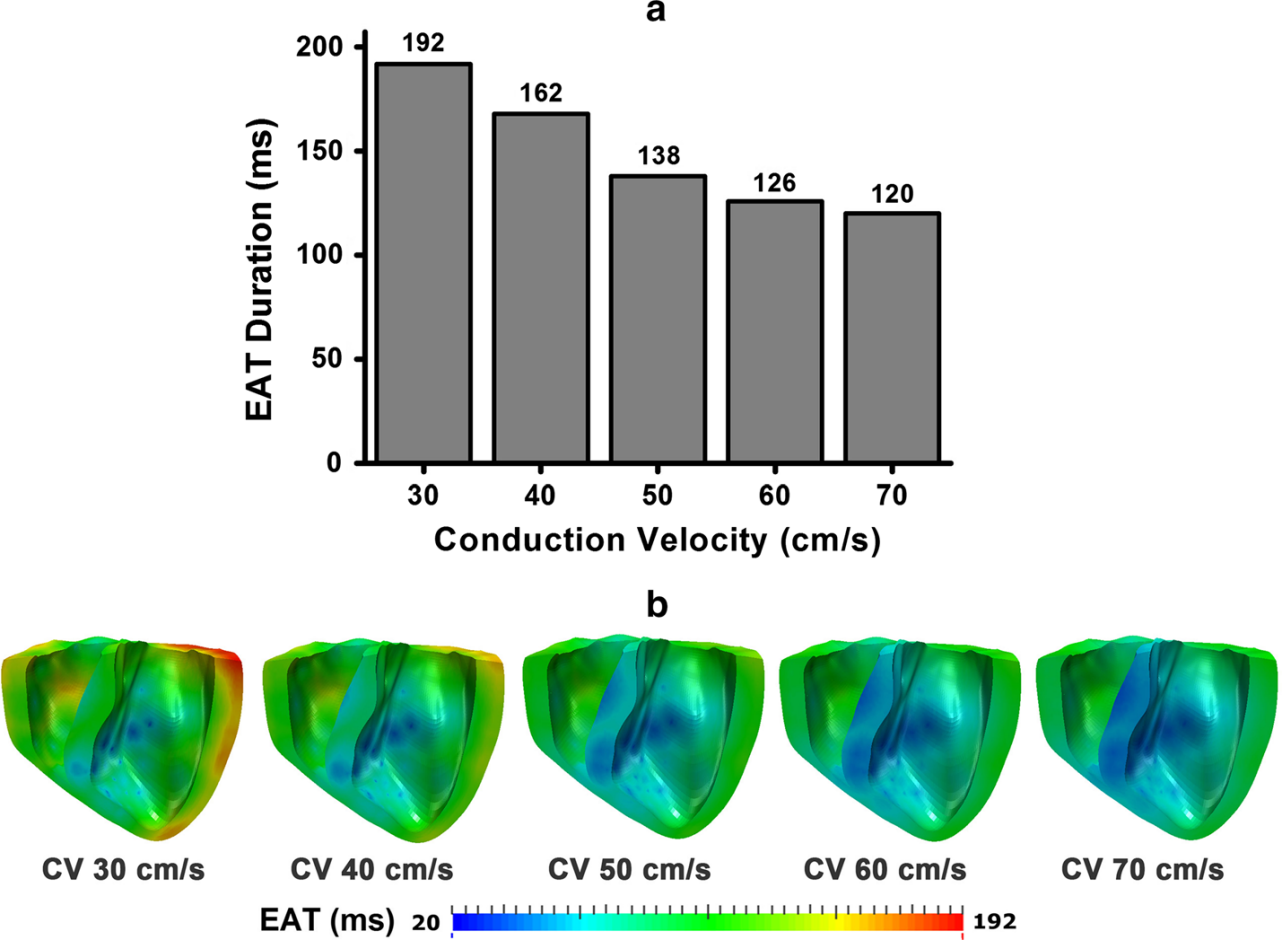
Electrical Activation Time (EAT). **a** Duration of EAT and **b** transmural distribution of EAT

Figure 3 shows the EAT duration, which was estimated from electrical simulation results under various MCV conditions. EAT duration gradually decreased as the MCV increased, as shown in Fig. 3a. The duration of EAT was 192 ms in 30 cm/s MCV case, 162 ms in 40 cm/s MCV case, 138 ms in 50 cm/s MCV case, 126 ms in 60 cm/s MCV case, and 120 ms in 70 cm/s MCV case. Figure 3b shows the transmural distribution of EAT under each MCV condition. Because Purkinje terminal node activated at 20ms, the lowest EAT was 20 ms under all MCV conditions. However, the longest EAT varied in conjunction with MCV variation.

### Hemodynamic responses

**Fig. 4:**
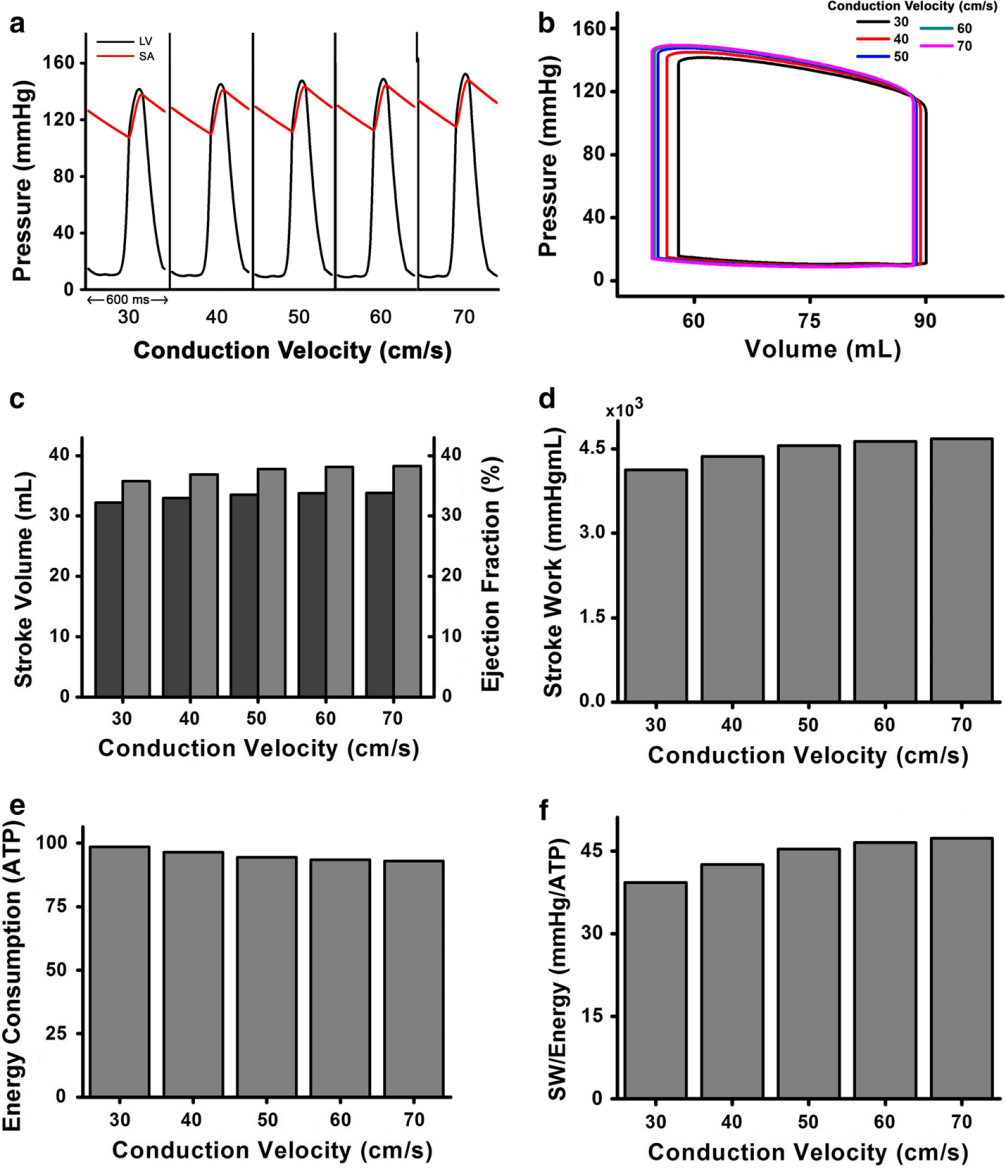
Cardiac mechanical responses. **a** Pressure waveform in the left ventricle (LV) and systemic artery toward time, **b** pressure–volume (PV) curves of LV, **c** stroke volume (SV) and ejection fraction (EF) of LV, **d** LV stroke work (SW), **e** energy consumption of LV in the form of ATP, and **f** SW over ATP

As shown in Fig. 4a, the left ventricle (LV) generated 150 mmHg systolic pressure and had a mean arterial pressure 145 mmHg under the highest MCV case of 70 cm/s. Meanwhile, it generated 141 mmHg systolic pressure and had a mean arterial pressure of 137 mmHg under the lowest MCV case of 30 cm/s. Based on the pressure–volume (PV) loop of LV (see Fig. 4b), the highest MCV condition of 70 cm/s had the smallest systolic and diastolic volumes (approximately 54 and 88.2 mL, respectively). Systolic and diastolic volumes of the LV increased with lower MCV, and were largest under the 30 cm/s MCV condition (approximately 58 and 90 mL, respectively).

Therefore, stroke volume (SV) and ejection fraction (EF) were the greatest (i.e., 34.2 mL and 38.3%, respectively) under the 70 cm/s MCV condition, were lowest (32 mL and 35.7%, respectively) under 30 cm/s MCV condition (see Fig. 4c). These results indicate that cardiac output is greater under higher MCV conditions. Accordingly, stroke work (SW), the area within the PV curves, was greater under the higher MCV conditions (Fig. 4d). However, ventricular ATP consumption rate was lower under higher MCV condition (Fig. 4e). Under 30 cm/s MCV, the ATP consumption was approximately 98.6 s^−1^, followed by the 40 cm/s MCV with 96 s^−1^, 50 cm/s MCV with 94 s^−1^, 60 cm/s MCV with 93 s^−1^, and 70 cm/s MCV with 92 s^−1^, respectively. The ATP consumption decreased by approximately 6% between these cases. Finally, SW over ventricular ATP consumption, which indicates energetic pumping efficiency of the ventricle, was smaller under higher MCV conditions (Fig. 4f), which indicates that the ventricle consumes less energy and does more work under higher MCV conditions. The index SW/ATP was greatest under the 70 cm/s MCV condition, with approximately 47 mmHg mL/ATP, followed by 46 mmHg mL/ATP under the 60 cm/s MCV condition, 45 mmHg mL/ATP under the 50 cm/s MCV condition, 42 mmHg mL/ATP under the 40 cm/s MCV condition, and 39 mmHg mL/ATP under the 30 cm/s MCV condition.

### Model validation

**Fig. 5:**
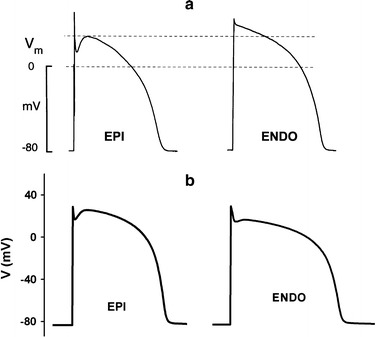
Action potential shape in the single ventricular cell. **a** From experimental data of Nabauer et al. [20], and **b** from our simulation

To validate the electrical properties in our model, we simulated the shape of AP in the single cell and estimated the MCV values in the ventricular tissue. Then, we compared our simulation result with the previous experimental studies. The simulated AP shapes in the single cell matched with the shape of AP in the experimental data from Nabauer et al. [20] as shown in Fig. 5. Next, we obtained the MCV 70 cm/s which was matched to the normal conduction velocity according to experimental data of Taggart et al. [18], by setting the cellular resistivity into 162 $\Omega$ cm.

## Discussion

In this study, we examined quantitatively the effect of myocardium electrical conductivity on cardiac mechanical performance using the existing 3D electromechanical representation of a failing ventricle [6–8]. We compared the cardiac electrical and mechanical responses under five different MCV conditions, from 30 to 70 cm/s with 10 cm/s interval during sinus pacing. The main findings of this work are as follows:

1. EAT is shorter under higher MCV conditions.
2. Ventricles under higher MCV conditions produces greater mechanical functions including better generation of intra-ventricular pressure, cardiac output, and stroke work.
3. ATP consumption rate is decreased under higher MCV conditions.

The concept of non-uniformity of a ventricle has a strong relationship with ventricular function and performance. The ventricle is not formed from a simple geometric shape, but consists of various fibers, which are oriented in different directions and with different diameter [16]. The ventricle fibers themselves are comprised of at least three electrically distinct cell types: epi-, mid-, and endo-myocardium cells. APD is the longest at the mid-myocardium [21], which make the depolarization wave lasts longest in this region (see Fig 3). The prolongation of mid-myocardium AP is facilitated by the delayed rectifier current ($I_{\rm{Ks}}$) being smaller and having a slower activation while exhibiting a larger late sodium current (late $I_{\rm{Na}}$) and sodium-calcium exchange current ($I_{\rm{Na,Ca}}$) [21].

The ventricle depolarization timing is shorter with the higher MCV. According to equation (6) in the method section, MCV is inversely proportional with the time. This is why increasing the MCV will decrease the activation time, and conversely, reducing MCV will increase activation time, when considering the same distance (d). This phenomenon described by Fig. 3a that the EAT duration is gradually decreased with the increasing MCV value.

The cardiac systole occurs in response to the spontaneous electrical conduction of the whole ventricles. Rapid and organized conduction is necessary to generate efficient pressure during systole to pump blood out of ventricles. Our results showed that the pressure of left ventricle (LV) during systole is higher under faster MCV (see Fig. 4a). It describes that LV has more forces to pump blood out through circulation with faster MCV. However, the systolic and diastolic pressure resulted in this study is more than 120 and 80 mmHg, respectively, for all MCV cases, because we simulated using failing ventricle model with hypertensive condition. As we mentioned before in the Methods section, that we modeled the failing ventricle condition by decreasing 10% compliances of vascular system, so that the arterial blood pressure increased from normal condition. To best to our knowledge, the normal systolic pressure is less than 120 mmHg, and normal diastolic pressure is less than 80 mmHg (American Heart Association).

 SV is calculated by subtracting the end systolic volume (ESV) from end diastolic volume (EDV) in the PV curves. Both ESV and EDV of the LV are reduced under higher MCV. The ESV reduced by approximately 1.5%, whereas the EDV reduced by approximately 0.5%. The reduction in the ESV is higher than in the EDV, thereby forming a horizontally extended PV curves, generating larger SV values. Subsequently, EF is calculated by dividing the SV by the EDV of the LV in one cycle contraction. EF also increased under higher MCV conditions. Increasing in SV and EF (Fig. 4c) indicates that the LV ejects more blood with higher MCV. Consequently, SW, which means the amount of work done by ventricle during one cycle, is increased under higher MCV (see Fig. 4d). In contrast, the ventricular ATP consumption rate is decreased in response to the increasing in the MCV value (Fig. 4e). This indicates that the ventricle consumes less energy to pump out the blood with higher MCV. Higher MCV values allow the regions of ventricle to contract more synchronously than the lower MCV values, due to the larger wavelengths. Synchronous contraction helps the ventricle to pump out more blood. This synchronization explains why the ATP consumed by a ventricle with higher MCV values is smaller than that of a lower MCV. These results explicate that the ventricular pumping activity is more efficient under higher MCV by consuming smaller energy (ATP) while carrying out more works (Fig. 4f).

In conclusion, this study reveals that MCV has strong correlation with the cardiac pumping efficacy. The alteration of MCV affects to the electrical and mechanical behavior of the heart. The obtained results provide useful information to estimate the effect of MCV on the electro-physiology and hemodynamic responses of the ventricle and can be used for further study about arrhythmogeneis and heart failure.

There are several limitations of the present study. First, we did not conduct experimental or clinical data in the study. Instead, we used the validated cell model and methodologies from previous studies [6–8, 12, 13]. For cardiac electrophysiology, we implemented human ventricular cell model from ten Tusscher et al. [12] which has been already validated with experimentally measured data [18]. For cardiac mechanics model, we applied the myofilament dynamics model from Rice et al. [13]. Next, we used the computational model of a failing canine ventricle, which have different mechanical characteristic than that of a failing human ventricles. We also only implemented one way EC Coupling model in this study, so that the cardiac mechanical activity can not affect to the electrophysiological behavior of the heart. Although in fact, such phenomena could occur physiologically. Next, the lumped model of the circulatory system was used to reduce the complexity of the model. Additionally, engaging electromechanical delay in further study might improve the understanding about the effect of MCV to the cardiac mechanical behavior. However, these potential limitations are not expected to influence our conclusion significantly.

## Clinical implications

Over the past 3 decades, the clinical, experimental, and theoretical studies have validated that slow conduction, plays important role in the pathogenesis of cardiac arrhythmias. Slowed myocardial conduction can increase the probability of the occurrence of cardiac arrhythmia through the formation of slow conducting re-entry circuits [1]. However, Not only arrhythmia, the reduction of myocardium conduction velocity also had been widely linked to the pathogenesis of heart failure such as fibrosis and hypertrophy. Several studies have validated that fibrosis induced the reduction of myocardium conduction velocity, which further cause the impaired cardiac function. Therefore, knowing how far the reduction in conduction velocity affect to the pumping efficacy would be valuable to study the pathogenesis of the fibrosis and hypertrophy.

## Conclusions

Based on these findings, we conclude that the cardiac pumping efficacy is better under higher MCV conditions due to an increasing in the force generation during systole and cardiac output while consuming less energy.

## Abbreviations

AP: action potential
APD: action potential duration
EAT: electrical activation time
EC: excitation–contraction
EDV: end diastolic volume
EF: ejection fraction
ESV: end systolic volume
LV: left ventricle
MCV: myocardial conduction velocity
PV: pressure–volume
SR: sarcoplasmic reticulum
SV: stroke volume
SW: stroke work
